# Effects of a Virtual Reality Intervention on Women’s Menstrual Problems Related to Endocrine Disruptors: A Randomized Controlled Repeated-Measures Pilot Study

**DOI:** 10.3390/healthcare14111583

**Published:** 2026-06-04

**Authors:** SoMi Park, Yun Jeong Hwang, ChaeWeon Chung

**Affiliations:** 1 Wonju College of Nursing, Yonsei University, Wonju 26426, Republic of Korea; somi@yonsei.ac.kr (S.P.); hwangyj@yonsei.ac.kr (Y.J.H.); 2 The Research Institute of Nursing Science, College of Nursing, Seoul National University, Seoul 03080, Republic of Korea

**Keywords:** virtual reality, dysmenorrhea, endocrine disruptor, premenstrual syndrome, young women

## Abstract

Background: Menstrual disorders are among the most common health problems faced by young women, yet effective interventions remain limited. Recent evidence has linked endocrine disruptors (EDs) to dysmenorrhea and premenstrual syndrome (PMS), suggesting that reducing exposure may alleviate symptoms. Purpose: The aim of the study was to investigate the effects of an immersive virtual reality (VR) intervention designed to promote protective behaviors against EDs and to evaluate its longitudinal impact on menstrual pain and PMS among young adult women. Methods: A nonequivalent comparison group pretest and repeated posttest experimental design was applied, using a convenience sample of 30 participants. Guided by the Information–Motivation–Behavioral skills model, the immersive VR intervention incorporated educational content, motivational cues, and avatar-based play experiences to enhance knowledge, perceived benefits, and self-efficacy. The comparison group received a small-group education session. Data were collected at baseline and at 4, 8, 12, and 24 weeks post-baseline. Results: Repeated-measures revealed significant interaction effects between group and time for menstrual pain (F = 2.67, *p* = 0.039), perceived benefits of protection from ED exposure (F = 4.41, *p* = 0.003), self-efficacy in reducing ED exposure (F = 5.42, *p* = 0.001), and protective behaviors against EDs (F = 4.68, *p* = 0.002). However, the overall group-by-time interaction effect for PMS was not statistically significant (F = 2.05, *p* = 0.097). Conclusion/Implication for Practice: Immersive VR as part of digital interventions has the potential to transform patient education by enhancing engagement while promoting protective health behaviors and improving associated health outcomes. Future research should explore strategies to improve long-term behavioral adherence and examine whether booster sessions can help sustain the effects of the intervention over time.

## 1. Introduction

Menstrual disorders are among the most common health issues affecting young women [1], with more than 75% to 77% experiencing problems related to menstruation [2]. The most frequent complaint is menstrual pain, or dysmenorrhea [3]. Additionally, 39% to 52% of young women experience stress related to PMS, with common symptoms including headache (45%), back pain (68.3%) [4], and emotional disturbances that may begin up to a week before menstruation and persist for a week afterward [5]. Despite this prevalence, the rate of seeking medical care for menstrual problems remains low. Instead, many young women rely on painkillers for temporary symptom relief rather than pursuing effective treatments [6]. Menstrual issues are influenced by physiological factors such as body mass index (BMI), age at menarche, family history, sleep patterns, diet, and contraceptive use [7]. Psychosocial factors including self-esteem, depression, and attitudes toward menstruation, as well as lifestyle factors such as exercise, smoking, alcohol use, physical activity, and stress, are also associated with menstrual problems [8,9].

Menstrual problems have also been linked to exposure to endocrine disruptors (EDs) [10,11]. Humans are exposed to EDs through multiple pathways, including dietary intake, dermal absorption from personal care products and cosmetics, inhalation, and environmental contact [12]. EDs with estrogenic properties, such as alkylphenols, bisphenol A, and phthalates, have been shown to trigger precocious puberty [13] and increase the risk of estrogen-dependent conditions, including endometriosis, uterine fibroids, premature ovarian failure, and polycystic ovary syndrome [10,14]. Beyond the reproductive system, EDs affect immune function. They interfere with cytokine and immunoglobulin synthesis, modulate inflammatory mediator activity, and alter immune cell activation and survival, thereby disrupting the body’s natural immune response [15]. Such dysregulation enhances uterine inflammation, a primary cause of menstrual pain, leading to more severe symptoms.

Human exposure to EDs occurs mainly through everyday products, including plastic bottles, food containers, can liners, toys, cosmetics, detergents, and agricultural pesticides [16]. Dietary intake is the predominant route of exposure, accounting for more than 90% of total chemical ingestion [17]. Accordingly, recent studies have emphasized dietary modification interventions to reduce ED exposure by substituting household or personal products [18,19]. However, some interventions have shown limited effectiveness, possibly because the duration was insufficient to influence motivational factors [20] or because inadequate product labeling hindered participants from identifying ED-free foods [18]. These findings highlight the need for better-designed dietary interventions in future research.

Meanwhile, prior interventions using avatars in VR have consistently influenced health-related outcomes in young people [21], with demonstrated benefits for motivation [22] and self-efficacy [23]. For instance, adolescents with cancer who underwent VR-based simulations of treatment procedures reported improved pain relief and greater self-efficacy for pain management [24]. Similarly, young adults with congenital heart disease demonstrated enhanced understanding of their condition following VR education [25]. Such outcomes occur because users readily identify with avatars, experience immersion, feel connected to them [21], and recognize emotions expressed through avatars [26]. Given the invisible nature of EDs, VR provides an effective platform to visually depict bodily changes caused by exposure. Importantly, prior intervention studies have largely examined VR only in terms of behavioral outcomes; thus, this study sought to assess both protective behaviors against EDs and their longitudinal health effects on menstrual pain and PMS among young adult women.

### Conceptual Framework

**Figure 1:**
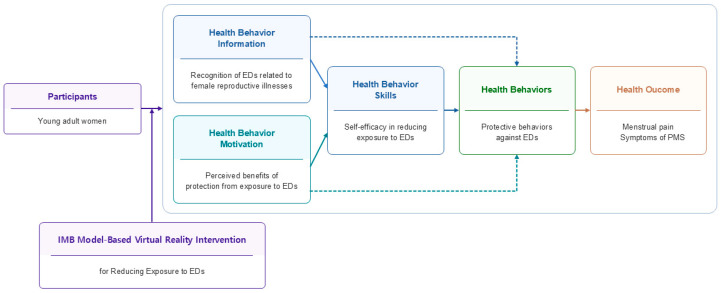
Conceptual framework of the study.

The conceptual framework of this study was based on the information–motivation–behavioral skills (IMB) model [27], which organizes the fundamental determinants of health behaviors: information, motivation, and behavioral skills (Figure 1). Compared with the Health Belief Model [28] and the Theory of Planned Behavior [29], which offer valuable insights into perceived risks and social norms, the IMB model uniquely emphasizes behavioral skills as a critical mediator between information, motivation, and actual behavior change. This emphasis is particularly relevant to ED exposure prevention because it addresses the complex “knowledge-to-action” gap in environmental health contexts. The IMB model was therefore considered appropriate for this study because it enabled the integration of an immersive VR intervention in which participants could practice and reinforce essential behavioral skills in a controlled, realistic environment.

First, within the IMB model, health behavior information is a prerequisite for risk-reduction behaviors and includes the knowledge necessary for individuals to adopt change. In this study, information on ED-related female reproductive illnesses was provided to young adult women to increase their knowledge of EDs. Second, health behavior motivation encompasses attitudes, social norms, and perceived costs and benefits of prevention. Accordingly, this study emphasized the perceived benefits of avoiding ED exposure. Understanding the advantages of reducing ED exposure can strengthen motivation and, in turn, positively influence young women’s choices to adopt protective behaviors. Third, health behavior skills involve not only objective abilities but also perceived self-efficacy in performing health-related actions. Thus, this study sought to enhance self-efficacy for reducing ED exposure. According to the model, when adequate information and strong motivation are present, self-efficacy—the behavioral skill necessary to effectively carry out health behaviors—is enhanced. Furthermore, role-playing positive health outcomes that result from protective behaviors has been shown to foster essential behavioral skills and improve self-efficacy. In this study, participants used avatars in VR to choose foods that minimized ED exposure and to indirectly experience improvements in bodily health, thereby reinforcing their behavioral skills.

In summary, this study aimed to increase knowledge about EDs through accurate information, improve motivation for protective behaviors by educating participants on the perceived benefits of avoiding ED exposure, and strengthen self-efficacy through VR-based role-play experiences with avatars. Ultimately, the intervention was intended to reduce menstrual pain and PMS symptoms by encouraging health behaviors that lower exposure to EDs.

## 2. Methods

### 2.1. Study Design

This study employed a randomized controlled repeated-measures design. The use of a quadratic posttest enabled the researchers to examine patterns of change in the effects of the intervention. The study hypothesized that the intervention group would show greater improvements in menstrual pain and PMS across the study period (4, 8, 12, and 24 weeks post-baseline) compared with the comparison group.

### 2.2. Participants

A convenience sample of 30 young adult women was recruited in June 2023 through social networking services such as Facebook and Instagram from the W city community, South Korea. The inclusion criteria were: (1) unmarried women, (2) aged 19–20 years, (3) with a BMI in the normal range (18.5 ≤ BMI < 23 kg/m^2^), (4) regular menstrual cycles for at least the past six months, and (5) voluntary agreement to participate. Because both obesity and underweight are known to be associated with menstrual problems, women outside the normal BMI range were excluded. Women taking oral contraceptives or psychiatric medications were also excluded.

Sample size was determined to evaluate the effects of the immersive VR intervention on both groups over time (baseline and at 4, 8, 12, and 24 weeks). Using G*Power 3.1.9.4 for repeated-measures ANOVA, with α = 0.05, power = 0.90, and an effect size of 0.25, the required sample size was 26. To account for a 15% attrition rate, recruitment continued until 30 participants (15 per group) were enrolled.

Potential participants expressed interest through phone, email, or text message as listed on the online recruitment flyer. Those meeting the inclusion criteria provided written consent by email and were randomly allocated by the Research Randomizer (version 4.0) to the experimental group or the comparison group accordingly. Anonymity was maintained across allocation, pretest, and posttests. One participant in the comparison group withdrew at 12 weeks due to personal reasons, leaving a final sample of 29 for analysis.

### 2.3. Study Groups

#### 2.3.1. The Immersive VR Intervention Group

**Figure 2:**
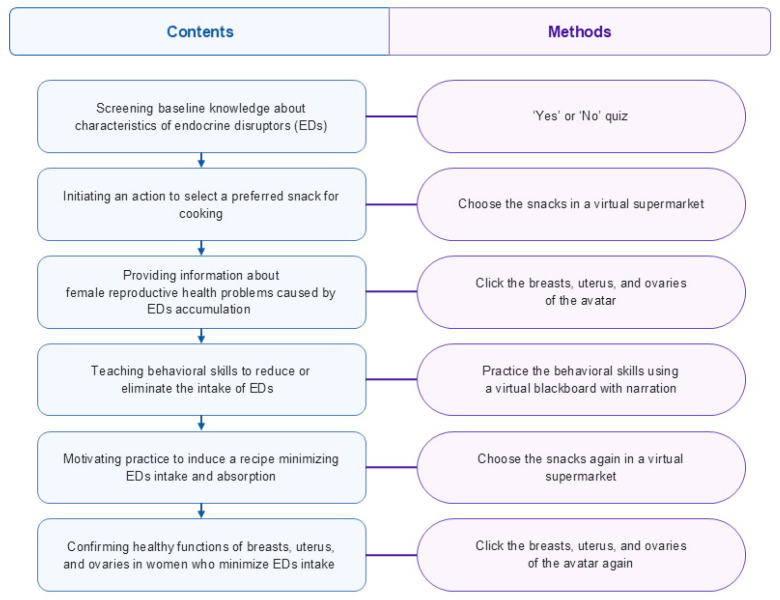
Immersive VR intervention flow based on the IMB model.

The immersive VR intervention, developed by the research team in a prior study phase, was delivered using the Oculus Quest 2 head-mounted display (HMD) (Meta Platforms, Inc., Menlo Park, CA, USA) (Figure 2) [30]. The intervention design was guided by the Information–Motivation–Behavioral (IMB) Skills Model, and the algorithm and scenario composition are described in detail in the VR program development study [30].

The intervention involved 15 participants divided into three groups of five. A research assistant scheduled sessions by sending text messages with three available dates within a week. Participants confirmed appointments via phone the day before, and groups were arranged accordingly.

The intervention was conducted in a lecture room at the university with which the principal investigator was affiliated. The environment was safe and spacious, accommodating five participants at a time. The intervention was led by a master’s-trained nurse (A) with research experience in educational interventions on EDs. Participants were informed that they could immediately remove the HMD if they felt nausea, anxiety, or any discomfort. They were also told that they could terminate the session at any time without needing to provide a reason. To prevent cyber-sickness, each session was limited to 15 min. Throughout the experience, participants remained seated in a comfortable position to reduce the risk of injury due to impaired vision while wearing the HMD. Before the intervention, participants’ baseline familiarity with VR technology was assessed; none had prior experience with immersive VR. Each participant received VR equipment and practiced using it. To systematically monitor potential cybersickness, the researcher conducted structured verbal screening for common symptoms, such as dizziness, nausea, and visual fatigue, immediately after each 15 min session. The sessions proceeded smoothly, with no reported cybersickness or adverse events, and no participants chose to terminate a session early.

#### 2.3.2. Comparison Group with a Small Group Lecture

The comparison group consisted of 15 participants, divided into three groups of five. Each group was informed of the time and location for their session by the research assistant. The educational sessions included a 15 min lecture followed by a 5 min Q&A. PowerPoint materials covered EDs, female reproductive health problems associated with ED accumulation, and behavioral skills to reduce or eliminate ED intake. As part of compensatory equalization of treatment, outlined in the written informed consent, three participants who requested the opportunity to experience the immersive VR intervention were allowed to do so after data collection was completed at 24 weeks post-intervention. The interventionist for this group was a master’s-prepared nurse (B) with teaching experience. To prevent diffusion of intervention effects, the comparison group sessions were held one week before the immersive VR intervention sessions.

Each interventionist was informed only about the assigned intervention—either the immersive VR or the small group lecture. The principal investigator provided a standard protocol and trained both interventionists in the core elements of the study to ensure fidelity and consistency, and their demonstrations were verified prior to implementation.

### 2.4. Measurements

#### 2.4.1. Menstrual Pain

Menstrual pain was assessed using a single item: “How severe was the pain during your period last month?” Responses were rated on a 10-point scale ranging from 1 (not at all) to 10 (very severe). Higher scores indicated greater menstrual pain.

#### 2.4.2. Premenstrual Syndrome (PMS)

PMS is defined as “a condition characterized by a combination of physical, emotional, and behavioral symptoms that occur during the luteal phase of the menstrual cycle (approximately the two weeks before menstruation) and resolve with the onset of menstruation” [31]. PMS was measured with one item: “How severe were the discomforts of premenstrual syndrome in the two weeks before your period last month?” Responses were rated on a 10-point scale from 1 (not at all) to 10 (very severe). Higher scores reflected greater PMS severity.

#### 2.4.3. Recognition of EDs Related to Female Reproductive Illnesses

Recognition of ED-related illnesses was measured using six items adopted from Park and Chung [32]. Participants rated the likelihood that infertility, dysmenorrhea, endometriosis, miscarriage, breast cancer, and PMS are associated with EDs. A 4-point Likert scale ranging from 1 (very unlikely) to 4 (very likely) was used, with higher scores indicating stronger perceived associations. Content validity was confirmed by a gynecologist and two nurse researchers during development. Cronbach’s *α* was 0.81 in Park and Chung [32] and 0.91 in this study.

#### 2.4.4. Perceived Benefits of Protection from Exposure to EDs

Perceived benefits were measured with 11 items developed by Kim and Choi [33]. Each item asked participants to rate the potential benefits of avoiding ED exposure on a 5-point Likert scale (1 = very unlikely, 5 = very likely). Total scores ranged from 11 to 55, with higher scores reflecting greater perceived benefits. Cronbach’s α was 0.77 in Kim and Choi [33] and 0.89 in this study.

#### 2.4.5. Self-Efficacy for Reducing Exposure to EDs

Self-efficacy was measured using 17 items from the Self-Efficacy Scale by Sherer et al. [34], modified by Kim and Choi [33]. Items assessed confidence in performing behaviors to reduce ED exposure, rated on a 5-point Likert scale (1 = very unlikely, 5 = very likely). Possible scores ranged from 17 to 85, with higher scores indicating greater self-efficacy. Cronbach’s α was 0.93 in Kim and Choi [33] and 0.94 in this study.

#### 2.4.6. Protective Behaviors Against EDs

Protective behaviors were assessed using a 19-item instrument from Park and Chung [32]. Participants rated the frequency of ED-avoidance behaviors in daily life on a 4-point Likert scale (1 = never, 4 = always). Scores ranged from 19 to 76, with higher scores reflecting more protective behaviors. Content validity was established, with Cronbach’s *α* reported as 0.85 in Park and Chung [32] and 0.90 in this study.

### 2.5. Data Collection Method and Process

Data collection was conducted by three senior nursing students who volunteered for the study and were trained by the principal investigator on the study schedule and procedures. To minimize potential performance and detection bias, the data collectors received standardized training to ensure consistency in their responsibilities. Their role was limited to scheduling, technical support, and protocol-adherence monitoring; they were not involved in intervention delivery or outcome interpretation.

Data were collected five times: at baseline and at 4, 8, 12, and 24 weeks post-intervention. Structured self-administered questionnaires were used to assess intervention effects over time. Each data collector was assigned responsibility for five participants from both the immersive VR group and the comparison group. Assignments remained unchanged throughout the study. The data collectors also served as liaisons with the principal investigator to address participant questions or concerns. Because participants completed responses directly without data collectors’ involvement, this procedure substantially reduced the possibility of detection bias.

**Figure 3:**
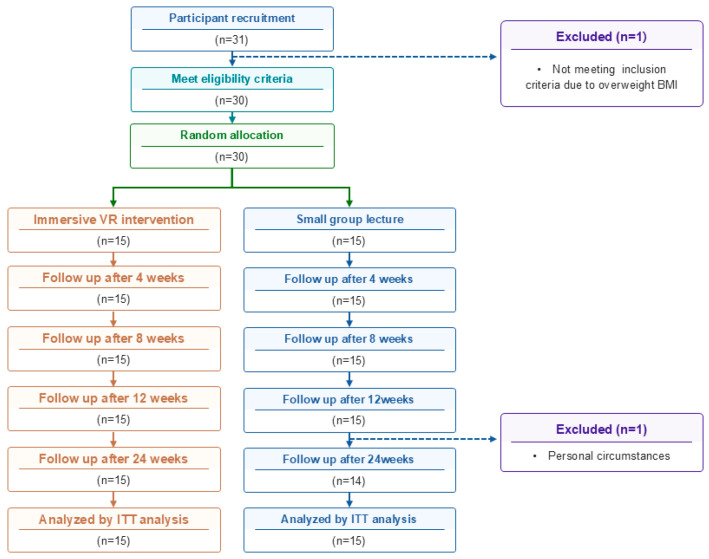
Research flow diagram.

Baseline data were collected before the interventions, and completion of the self-administered questionnaire required approximately 15 min. Follow-up data were collected online by each assigned research assistant. All responses were double-coded using case numbers to maintain confidentiality. As a token of appreciation, participants received a tumbler valued at $20 after completing the posttest (Figure 3).

### 2.6. Statistical Analyses

(1) Normality tests for the general characteristics of the intervention and comparison groups, as well as study variables, were conducted using the Shapiro–Wilk test. Results indicated that menstrual cycle, perceived benefits of protection from ED exposure, self-efficacy in reducing ED exposure, and protective behaviors against EDs met the assumption of normality (W = 0.86–0.97, *p* = 0.086–0.679). However, age, menstrual pain, and PMS symptoms did not meet the normality assumption (W = 0.70–0.85, *p* = 0.011–0.044).
(2) Descriptive statistics for the general characteristics and study variables of both groups were calculated using mean and standard deviation or median and interquartile range, depending on the results of the normality test. Pre-homogeneity between groups was examined using either the independent *t*-test or the Mann–Whitney U test, according to distributional assumptions.
(3) Differences in the six study variables between the immersive VR intervention and comparison groups over time were tested using a generalized linear mixed model (GLMM). Differences in changes between specific time points were examined with the Mann–Whitney U test.
(4) To avoid overestimating outcomes by excluding dropouts, an intention-to-treat (ITT) analysis was performed. Missing values for the participant (n = 1) who withdrew were replaced using mean imputation [35].

### 2.7. Ethical Issues and Approval

Approval for this study was obtained from the Institutional Review Board (No. CR323035) prior to commencement. The study was also registered with the Clinical Research Information Service of the Korea Disease Control and Prevention Agency (http://cris.nih.go.kr/cris/index/index.do, accesses on 3 June 2024) (Trial Registration No. KCT0009630, 7 October 2024).

All participants received both verbal and written explanations of the study’s purpose, objectives, and procedures. They were assured that withdrawal was permitted at any time without penalty. Prior to signing written consent forms, participants were informed that participation was voluntary, all data would be anonymized, and personal information would remain confidential. Additionally, volunteers who did not meet the BMI inclusion criteria were informed of the study purpose and inclusion restrictions. They were later given the opportunity to participate in the immersive VR intervention after study completion, coinciding with the post-study session provided to the comparison group.

## 3. Results

### 3.1. Homogeneity Test

The general characteristics of age (z = −0.30, *p* = 0.77) and menstrual cycle (z = 1.78, *p* = 0.09) were homogeneous between the immersive VR intervention and comparison groups. The study variables of the two groups also demonstrated homogeneity across all measures, including menstrual pain (z = −1.34, *p* = 0.18), PMS (z = −1.39, *p* = 0.17), recognition of ED-related female reproductive illnesses (z = −0.92, *p* = 0.36), perceived benefits of protection from exposure to EDs (t = −0.31, *p* = 0.76), self-efficacy in reducing exposure to EDs (t = −1.31, *p* = 0.20), and protective behaviors against EDs (t = 0.02, *p* = 0.99). All *p*-values were above the conventional significance threshold of 0.05, supporting the assumption of baseline homogeneity between groups.

### 3.2. Effects of the Immersive VR Intervention

The effects of the immersive VR intervention are summarized in Table 1.

Repeated-measures analysis revealed a significant interaction effect between group and time for menstrual pain (F = 2.67, *p* = 0.039) while the interaction effect for PMS was not significant (F = 2.05, *p* = 0.097).

Over time, significant differences were also observed in menstrual pain (F = 8.22, *p* < 0.001) and PMS (F = 7.67, *p* < 0.001). However, in group differences, no significant differences were found between the groups for menstrual pain (F = 0.04, *p* = 0.843) or PMS (F = 0.00, *p* = 0.960).

Significant interaction effects between group and time were also observed for perceived benefits of protection from ED exposure (F = 4.41, *p* = 0.003), self-efficacy in reducing ED exposure (F = 5.42, *p* = 0.001), and protective behaviors against EDs (F = 4.68, *p* = 0.002). Time effects were significant for perceived benefits of protection from ED exposure (F = 5.78, *p* < 0.001), self-efficacy in reducing ED exposure (F = 32.53, *p* < 0.001), and protective behaviors against EDs (F = 23.35, *p* < 0.001). A significant group difference was identified only for protective behaviors (F = 11.43, *p* = 0.002).

In contrast, for recognition of ED-related female reproductive illnesses, a significant effect was observed only for changes over time (F = 12.70, *p* < 0.001). Neither the group difference (F = 3.78, *p* = 0.062) nor the interaction effect (F = 0.78, *p* = 0.541) reached statistical significance.

### 3.3. Differences in Changes in Menstrual Pain and PMS at Each Time Point Between the Two Groups

**Figure 4:**
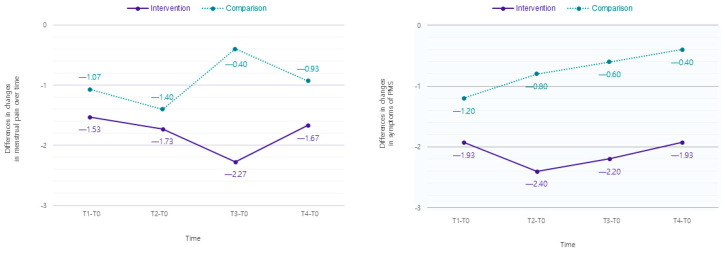
Changes in menstrual pain and PMS over time.

The patterns of change in menstrual pain and PMS between the immersive VR intervention and comparison groups at each time point are shown in Figure 4. In the intervention group, menstrual pain demonstrated a gradual decrease, forming a consistent downward trend through 4, 8, and 12 weeks post-intervention. At 24 weeks, however, the trend shifted upward, suggesting a slowing in the reduction in pain, though levels remained below baseline. By contrast, the comparison group showed decreases in menstrual pain up to 4 and 8 weeks, followed by an upward trend beginning at 12 weeks, indicating a recurrence of pain thereafter. The difference in changes between the two groups was statistically significant at 12 weeks post-intervention (z = −3.14, *p* = 0.002).

For PMS, the immersive VR intervention group experienced reductions in symptoms up to 4 and 8 weeks after the intervention, with scores forming a downward pattern. From 12 weeks onward, however, PMS scores increased, although they remained below baseline levels. The comparison group also reported symptom relief immediately following the small-group lecture, but PMS scores rose steadily from 4 through 24 weeks post-intervention. Between-group differences were statistically significant at 8 weeks (z = −2.32, *p* = 0.021), 12 weeks (z = −2.21, *p* = 0.027), and 24 weeks (z = −2.02, *p* = 0.043) post-intervention.

## 4. Discussion

Although recent evidence highlights the harmful effects of EDs on women’s reproductive health [10,14], few interventions have been actively developed to address common menstrual problems beyond pharmacological approaches such as pain medication. As modern environments increase both exposure to and accumulation of EDs in women’s bodies [10], raising awareness of their adverse effects and promoting practical health behaviors to reduce exposure are essential. This study was therefore designed to alleviate menstrual pain and PMS by encouraging adherence to protective behaviors that reduce or eliminate ED intake, implemented through an immersive VR intervention. Beyond demonstrating intervention effects, this study also revealed the value of immersive VR’s sense of presence, vivid presentations, and interactivity [36], which made it especially suitable for targeting the invisible nature of EDs. Furthermore, the intervention was grounded in the IMB model [27], ensuring that each component—information, motivation, and behavioral skills—directly contributed to protective health behaviors and health outcomes. In particular, improvements in self-efficacy were found to mediate the translation of knowledge and motivation into behavioral skills in this study.

Because enhancing motivation is a well-established strategy for achieving behavioral goals, the intervention was designed to simulate a virtual supermarket where participants could shop for snacks prepared using various cooking methods, thereby encountering realistic scenarios of everyday ED exposure. Avatar facial expressions provided real-time alerts regarding ED-related risks, while visual cues such as twinkling effects on the avatar’s body illustrated potential health issues associated with ED accumulation. Supplementary images and text-based information were also accessible via clickable content, offering opportunities for deeper learning about health consequences of ED exposure. These interactive features were intended to strengthen motivation by encouraging participants to choose alternatives that would minimize ED intake. Moreover, psychological identification with the avatar may have further reinforced motivation, supporting sustained engagement in protective health behaviors.

Self-efficacy, defined by Bandura [37] as the belief in one’s ability to execute behaviors necessary to achieve specific tasks, was another central focus of this intervention. Self-efficacy in reducing ED exposure was enhanced through experiential learning activities such as selecting recipes and snacks in the virtual supermarket. Quizzes, positive reinforcement by smiling avatars for correct answers, and the awarding of a virtual certificate likely contributed to improvements in participants’ self-efficacy. By lowering perceived barriers to enacting protective behaviors in daily life, these features appeared to foster greater engagement. Indeed, immersive VR interventions have been shown to strengthen self-efficacy across various domains, such as tracheostomy care training for healthcare providers and caregivers [38] and disaster preparedness for flood evacuation [39]. These findings are consistent with the broader framework of digital rehabilitation and behavioral medicine, which recognizes immersive VR as a robust platform for clinical symptom management and cognitive-behavioral reconstruction [40,41]. By transforming traditional, passive patient education into an active experiential process, VR-based interventions may enhance patient engagement and treatment adherence, thereby optimizing clinical outcomes in nonpharmacological symptom care.

Adherence to protective behaviors against ED exposure was higher in the immersive VR group compared to the small-group lecture approach. The VR intervention focused on minimizing ED accumulation in the body by providing virtual classroom experiences with clickable content. Specific actions emphasized included reducing consumption of instant foods and limiting the use of disposable products, plastics, sprays, and shampoos, thereby reinforcing daily life behaviors to reduce ED exposure. Personalized feedback -recognized as an effective strategy for behavior change [42]- was incorporated by allowing participants to self-manage their learning pace and repeat narrated text displayed on a virtual blackboard, which likely increased the impact compared with traditional lecture-based instruction. Additionally, the intervention content on instant foods was drawn from participants’ preferred food lists identified in a prior study [30]. This cultural tailoring aligns with evidence showing that diabetes self-management education and support programs adapted to cultural needs are more effective for minority populations [43]. Therefore, healthcare professionals should design interventions that reflect the preferences and cultural contexts of the target population to maximize effectiveness.

In particular, the immersive VR intervention was effective in reducing menstrual pain, with significant interaction effects between group and time. Many intervention studies have tested longitudinal effects up to 12 weeks, including dietary modification targeting menstrual pain and urinary BPA [11], action-guided interventions addressing optimistic bias about reproductive health problems and improving protective behaviors against EDs [32], and nurse-led online education programs for pain, anxiety, and quality of life in patients with irritable bowel syndrome [44]. Building on this, the present study sought to examine whether behavioral changes would persist beyond 12 weeks. Results showed that reductions in menstrual pain and PMS relapsed by 24 weeks post-intervention. While dietary interventions have demonstrated significant improvements in pain and quality of life for women with endometriosis sustained over six months [45], future trials should focus on strategies to optimize adherence to protective health behaviors against EDs. These may include controlling for potential confounders, as well as incorporating booster or re-education sessions to maintain long-term effects. The attenuation of intervention effects at 24 weeks underscores the need for long-term maintenance strategies, such as periodic VR booster sessions or integration with mobile health interventions, to counteract the fading of behavioral reinforcement over time.

Contrary to expectations, significant effects were not observed for recognition of EDs related to female reproductive illnesses or for PMS. This may be explained by the fact that EDs, environmental hormones, and female reproductive organs are often perceived as rare or unfamiliar topics among the general public. Thus, participants in both groups may have benefited equally from the educational content. Moreover, no significant group × time interaction was found for PMS. Because PMS typically occurs in the two weeks prior to menstruation, but participants were asked to evaluate symptoms retrospectively after their periods ended, recall bias may have influenced the results. Given that PMS is a multifactorial condition encompassing physical, psychological, and behavioral symptoms, the intervention may not have been sufficiently sensitive to capture these complexities. Nevertheless, a previous study of nurses reported that ED exposure accounted for 25% of the variance in PMS severity, a statistically significant finding [46]. This highlights the need for future interventions to address the broad range of factors contributing to PMS.

Several limitations should be considered when interpreting these findings. Data on menstrual pain and PMS were measured using single items and did not meet the assumption of normality. Although the Mann–Whitney U test was used to evaluate differences between the intervention and comparison groups at each time point, the possibility of Type I error cannot be excluded. Future studies should consider using standardized multi-item instruments to improve measurement precision, sensitivity, and validity. The generalizability of the results may be limited by the small sample size used in the generalized linear mixed model and repeated-measures ANOVA, as well as by the restricted age range of the participants. The observed short-term improvements may also have been partly driven by a temporary novelty effect or placebo response associated with immersive VR technology, potentially inflating participants’ initial enthusiasm. Finally, although the researchers attempted to enhance internal validity by blinding data collectors to participants’ group assignments, the participants themselves were not blinded.

## 5. Conclusions

The findings of this study indicate that the immersive VR program positively influenced health behaviors aimed at reducing exposure to environmental hormones, thereby contributing to the prevention of reproductive health problems associated with EDs. Educational strategies and interventions using VR with self-identifying avatars hold promise as effective tools for health promotion and disease prevention.

## Figures and Tables

**Table 1:** Differences in study variables between the two groups at each time point (Intervention (n = 15), Comparison (n = 15)).

Time Characteristics	Group	Baseline	4 Weeks	8 Weeks	12 Weeks	24 Weeks	Source	F	*p*
		M ± SD							
Menstrual pain	Intervention	6.53 ± 1.96	5.00 ± 1.18	4.80 ± 2.01	4.27 ± 1.39	4.87 ± 2.07	Group	0.04	0.843
	Comparison	5.73 ± 2.02	4.67 ± 1.95	4.33 ± 2.44	5.33 ± 1.72	4.80 ± 2.18	Time	8.22	<0.001
							G × T	2.67	0.039
PMS	Intervention	6.87 ± 2.50	4.93 ± 1.79	4.47 ± 1.99	4.67 ± 1.84	4.93 ± 1.90	Group	0.00	0.960
	Comparison	5.80 ± 1.70	4.60 ± 1.99	5.00 ± 1.36	5.20 ± 1.52	5.40 ± 1.81	Time	7.67	<0.001
							G × T	2.05	0.097
Recognition of ED-related female reproductive illnesses	Intervention	19.67 ± 2.66	21.87 ± 2.03	22.07 ± 2.19	22.47 ± 2.26	22.53 ± 2.10	Group	3.78	0.062
	Comparison	18.80 ± 2.65	20.53 ± 2.10	20.80 ± 2.40	20.80 ± 2.60	20.20 ± 2.93	Time	12.70	<0.001
							G × T	0.78	0.541
Perceived benefits of protection from exposure to EDs	Intervention	42.33 ± 5.98	47.40 ± 4.97	46.27 ± 5.98	47.13 ± 5.53	47.33 ± 5.50	Group	2.94	0.097
	Comparison	42.93 ± 4.71	45.53 ± 5.21	44.47 ± 5.25	42.13 ± 4.85	41.53 ± 4.87	Time	5.78	<0.001
							G × T	4.41	0.003
Self-efficacy in reducing exposure to EDs	Intervention	59.13 ± 8.73	68.27 ± 7.78	68.40 ± 9.36	69.07 ± 10.67	59.20 ± 10.36	Group	2.46	0.128
	Comparison	63.60 ± 9.90	67.07 ± 9.68	65.40 ± 10.11	61.00 ± 8.78	46.20 ± 4.59	Time	32.53	<0.001
							G × T	5.42	0.001
Protective behaviors against EDs	Intervention	44.87 ± 8.96	56.40 ± 8.35	57.87 ± 6.92	58.60 ± 7.35	51.93 ± 6.66	Group	11.43	0.002
	Comparison	44.80 ± 10.39	51.47 ± 9.74	51.60 ± 9.72	47.53 ± 7.46	35.47 ± 6.83	Time	25.35	<0.001
							G × T	4.68	0.002

PMS = premenstrual syndrome; EDs = endocrine disruptors.

## Data Availability

The datasets used and analyzed during the current study are available from the corresponding author on reasonable request.
